# P-398. External Validation of the Korean National Healthcare-Associated Infections Surveillance System (KONIS): A Surgical Site Infection Module Report

**DOI:** 10.1093/ofid/ofae631.599

**Published:** 2025-01-29

**Authors:** Young Keun Kim, Kyoung-Ho Song, Su Hyun Kim, Hye Ran Jeong, Eunjeong Shin, Jeongsuk Song, Sook-Kyung Park, Sohee Jang

**Affiliations:** Yonsei University Wonju College of Medicine, Wonju, Kangwon-do, Republic of Korea; Seoul National University College of Medicine, Seoungnam-si, Kyonggi-do, Republic of Korea; Korea University Ansan Hospital, Ansan, Kyonggi-do, Republic of Korea; Wonju Severance Christian Hospital, Wonju, Kangwon-do, Republic of Korea; Korea Disease Control and Prevention Agency, Cheongju, Ch'ungch'ong-bukto, Republic of Korea; Korea Disease Control and Prevention Agency, Cheongju, Ch'ungch'ong-bukto, Republic of Korea; Korea Disease Control and Prevention Agency, Cheongju, Ch'ungch'ong-bukto, Republic of Korea; Korea Disease Control and Prevention Agency, Cheongju, Ch'ungch'ong-bukto, Republic of Korea

## Abstract

**Background:**

Validation of national surveillance data is necessary to identify methodological problems within the surveillance program, to help increase compliance and participation in the surveillance program, and to identify data quality issues.

**Table.**
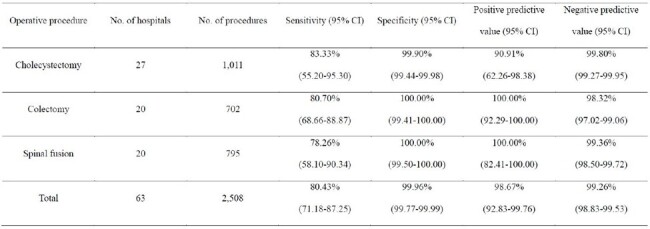
Validation of the data of cholecystectomy, colectomy and spinal fusion from Korean National Healthcare-associated Infections Surveillance System (KONIS): A Surgical Site Infection Module Report

**Methods:**

To determine the reliability and consistency of the application of surveillance definitions of the Korean Surgical Site Infections Surveillance System (KONIS-SSI), a validation study was conducted on data collected during the 4th quarter of 2022 for cholecystectomy, colectomy and spinal fusion. The 36,032 cholecystectomy of 162 hospitals, 11,819 colectomy of 60 hospitals and 14,949 spinal fusion of 57 hospitals were surveilled during 2022. Among them, a total of 2,508 operative procedures from 1,011 cholecystectomy of 27 hospitals, 702 colectomy of 20 hospitals and 795 spinal fusion of 20 hospitals were evaluated. Using the reviewers' classification of infection as the "gold standard", the sensitivity, specificity, positive and negative predictive value of the surveillance data were determined.

**Results:**

The sensitivity, specificity, positive predictive value and negative predictive value for surgical site infections was 80.43%, 99.96%, 98.67% and 99.26%, respectively. The rate of disagreement was 99.24%. The rate of agreement of cholecystectomy, colectomy and spinal fusion was 99.70% (95% CI 99.13-99.90, 1,008/1,011), 98.43% (95% CI 97.22-99.12, 691/702) and 99.37% (95% CI 98.54-99.73, 90/795), respectively. The reasons of disagreement were a disagreement of surgeons, lack of medical records and system instablity of each hospital.

**Conclusion:**

This study shows the necessity of continuous training and contact with surveillance personnel for maintaining data accuracy of surveillance data.

**Disclosures:**

**All Authors**: No reported disclosures

